# Preliminary Technical Validation of LittleBeats™: A Multimodal Sensing Platform to Capture Cardiac Physiology, Motion, and Vocalizations

**DOI:** 10.3390/s24030901

**Published:** 2024-01-30

**Authors:** Bashima Islam, Nancy L. McElwain, Jialu Li, Maria I. Davila, Yannan Hu, Kexin Hu, Jordan M. Bodway, Ashutosh Dhekne, Romit Roy Choudhury, Mark Hasegawa-Johnson

**Affiliations:** 1 Department of Electrical and Computer Engineering, Worcester Polytechnic Institute, Worcester, MA 01609, USA; 2 Department of Human Development and Family Studies, University of Illinois Urbana-Champaign, Urbana, IL 61801, USA; yannanh2@illinois.edu (Y.H.); kexinhu2@illinois.edu (K.H.); krwczyk2@illinois.edu (J.M.B.); 3 Beckman Institute for Advanced Science and Technology, University of Illinois Urbana-Champaign, Urbana, IL 61801, USA; 4 Department of Electrical and Computer Engineering, University of Illinois Urbana-Champaign, Urbana, IL 61801, USA; jialuli3@illinois.edu (J.L.); croy@illinois.edu (R.R.C.); 5 Research Triangle Institute, Research Triangle Park, NC 27709, USA; mariad@rti.org; 6 School of Computer Science, Georgia Institute of Technology, Atlanta, GA 30332, USA; dhekne@gatech.edu

**Keywords:** wearable devices, multimodal sensing, audio, electrocardiogram, inertial measurement unit, infants

## Abstract

Across five studies, we present the preliminary technical validation of an infant-wearable platform, LittleBeats™, that integrates electrocardiogram (ECG), inertial measurement unit (IMU), and audio sensors. Each sensor modality is validated against data from gold-standard equipment using established algorithms and laboratory tasks. Interbeat interval (IBI) data obtained from the LittleBeats™ ECG sensor indicate acceptable mean absolute percent error rates for both adults (Study 1, *N* = 16) and infants (Study 2, *N* = 5) across low- and high-challenge sessions and expected patterns of change in respiratory sinus arrythmia (RSA). For automated activity recognition (upright vs. walk vs. glide vs. squat) using accelerometer data from the LittleBeats™ IMU (Study 3, *N* = 12 adults), performance was good to excellent, with smartphone (industry standard) data outperforming LittleBeats™ by less than 4 percentage points. Speech emotion recognition (Study 4, *N* = 8 adults) applied to LittleBeats™ versus smartphone audio data indicated a comparable performance, with no significant difference in error rates. On an automatic speech recognition task (Study 5, *N* = 12 adults), the best performing algorithm yielded relatively low word error rates, although LittleBeats™ (4.16%) versus smartphone (2.73%) error rates were somewhat higher. Together, these validation studies indicate that LittleBeats™ sensors yield a data quality that is largely comparable to those obtained from gold-standard devices and established protocols used in prior research.

## 1. Introduction

Advances in personal sensing, Internet of Medical Things, and digital health have rapidly accelerated over the past decade [1,2,3,4], including the use of wearable devices among adults [2,5,6], children and adolescents [7,8,9], and infants [10,11,12,13]. Innovations in infant wearables, in particular, have predominantly focused on wireless skin-interfaced biosensors made of soft, flexible electronics that permit the continuous monitoring of vital signs, including but not limited to heart rate, blood pressure, temperature, respiration, and blood oxygen saturation (see [11,12,13]). Such sensors present notable benefits over more conventional wired systems, such as decreased iatrogenic effects (e.g., less damage to the infant’s delicate skin) and increased mobility (e.g., the infant can be picked up and held by a parent or caregiver, i.e., kangaroo care). Nonetheless, their development and testing have been largely restricted to neonatal and pediatric intensive care units (NICUs, PICUs), calling into question their utility and feasibility with respect to in-home monitoring under free-living conditions, in which infants’ movements, location, and environment may continuously change. Further, systems monitoring multiple vital signs typically require the placement of sensors on different parts of the body (see [11,12,14] for examples), which increases the complexity of the set up for the caregiver as well as the potential discomfort or restriction in movement for the infant, all of which may further decrease the feasibility for home use. Undoubtedly, these sensor systems address an important clinical need–to monitor the infant’s physical health via the detection of changes in vital signs in ways that are more patient-friendly. Understandably, however, these systems do not include sensing modalities, such as audio, that would permit the assessment of the infant’s biobehavioral development or social environment.

We aim to complement prior work on infant sensors for physical health monitoring in hospital settings by developing an infant wearable to monitor biobehavioral development and mental health in the home context. With this aim in mind, our interdisciplinary research team has developed a wearable platform, LittleBeats™, designed specifically for use with infants and young children. Compact and lightweight, LittleBeats™ is worn in the pocket of a specially designed shirt and integrates electrocardiogram (ECG), inertial measurement unit (IMU), and audio sensors on a single printed circuit board to permit daylong (8–10 h/day) remote assessments of infants/children and caregivers in home environments. Such wearable technology, especially when paired with machine learning algorithms, has the potential to transform our understanding of developmental processes through “big data” collected in real-world environments [15]. In this paper, we present the preliminary technical validation of the data quality of each LittleBeats™ sensor (ECG, IMU, audio) by assessing its signal quality in relation to the same signal obtained from an off-the-shelf gold-standard device. For this assessment, we compare the output performance of established algorithms and data reduction methods where the input signals are collected using the LittleBeats™ device and an off-the-shelf gold-standard device. In the subsections below, we first highlight the anticipated contribution of LittleBeats™ to the existing literature on wearable devices used with infants in home settings. Next, we identify the need for the current technical validation in the larger context of wearable sensor systems that comprise multiple parts and, thus, multiple validation steps. Finally, we present our specific objectives with respect to the technical validation of each sensor modality. 

### 1.1. Contribution of the LittleBeats™ Platform

Although advances in smartphone technology and wearable sensors have resulted in a surge of ambulatory assessments with adolescents and adults [16,17,18], current commercially available devices typically rely on one unit of analysis, such as self-reported behavior [18], physiological functioning [19,20,21], or audio recordings (EAR; [22] Language ENvironment Analysis [LENA], [23,24]), and almost none are feasible and/or validated for use with infants or young children. Indeed, the limited number of wearables that have been (a) used in the home (b) with infants and young children (c) across extended periods of time (e.g., daylong recordings) and (d) are validated is striking, particularly when compared with the proliferation of infant-wearable biosensors that have been designed for clinical use in hospital settings. The LENA system, which permits daylong audio recordings collected with an infant-worn recording device and includes proprietary software that automates the word counts of both infants and adults in the home, is a notable exception [23,24,25,26]. Commercially available infant wearables designed for home use that monitor physiological signals tend to lack rigorous validation (see [20,27]). Further, with respect to cardiac monitoring specifically, the quality signal of ECG makes it the gold standard compared with more noisy sensor signals used in wearables (e.g., phonocardiogram, photoplethysmography [28]). These latter sensors yield limited or gross cardiac measures (e.g., heart rate), whereas ECG data can capture a greater array of indices (e.g., cardiac vagal tone [29], which is assessed through R-R peak detection). With respect to monitoring infants’ physical movements, researchers have used arrays of IMUs, typically 3–4 sensors placed on infants’ limbs, to assess body position [30,31,32,33], and these studies complement a larger body of work using IMU and actigraphy to detect physical activity among preschool-aged children (see [34]). 

As seen above, infant wearables designed for in-home use have predominantly focused on a signal modality: audio, physiology, or motion. Prior work assessing a combination of behavioral (via audio, video, or motion sensors) and physiological (i.e., ECG, electroencephalography [EEG]) signals among infant samples in the home environment is extremely rare, and in these cases, separate data collection platforms or devices have been used to collect different data streams [15,35,36]. Such methods yield rich data, but limitations include the complexity of the sensor setup for parents to implement on their own, concerns about the child tolerating multiple sensors for prolonged periods of time, and challenges and pitfalls of post hoc signal synchronization. To the best of our knowledge, LittleBeats™ is the first wearable that focuses on simultaneously and continuously monitoring cardiac physiology, motion, and vocalizations of infants and young children. Given no existing device can simultaneously collect data from all three modalities, we see the gap and opportunity to develop this compact multimodal platform to capture the biobehavioral development and mental health of infants and young children from daylong recordings in the home context. 

### 1.2. Wearable Sensor Systems and Technical Validation as a Critical Step

Wearable sensor systems involve multiple steps, including (a) data acquisition, i.e., the collection of the raw sensor data, (b) data processing, i.e., the reduction of the raw sensor data into desired features and metrics, (c) health status detection, i.e., comparing reduced data or metrics against clinical thresholds for diagnostic or treatment purposes, (d) wireless communication, i.e., the transfer of data metrics and clinical information to physicians, parents, and/or other health professionals, and (e) power supply, which is an essential consideration underlying the successful implementation of all other parts of the system [10]. Each part of the sensor system, in turn, requires validation. As such, researchers have highlighted the need for digital health technologies, including wearables, to incorporate technical validation (or measurement verification, e.g., how do the raw signals compare to the technical gold standard?), analytic validation (e.g., how well do the algorithms applied to the raw sensor data yield meaningful measures of targeted behavioral or physiological constructs), clinical validation (e.g., how do the key measures perform in comparison with the clinical gold-standard?), and usability (or clinical utility, e.g., is the device easy to use? will the user use it in the intended way?) [37,38]. In this report, we undertake a critical step in the larger validation of the LittleBeats™ platform by conducting a technical validation on each type of sensor data acquired by the LittleBeats™ device. 

To this end, we use laboratory tasks and algorithms that have already been established and verified in the literature, and we compare their performance when applied to LittleBeats™ data versus data from a gold-standard device. We use established tasks and algorithms in this way to eliminate any uncertainty or performance bias that may be introduced by tasks or algorithms developed specifically for LittleBeats™. Relatedly, although we have designed the LittleBeats™ platform for use with infants and young children, the technical validation studies in this report are conducted primarily with adult samples because there are few established algorithms that are validated among infants/children. Furthermore, there are few if any standardized assessment protocols of physical movement (to evaluate IMU data) or vocalizations (to evaluate audio data) among infants and young children given the logistical challenges and impracticality of such procedures. The assessment of stress physiology is a notable exception, and we leverage a laboratory task (Still Face Paradigm [39,40,41]) and algorithm (Porges–Bohrer [41,42]), both of which are widely used and validated among infant samples (see Study 2). 

In tandem with the technical validation studies reported here, we have collected LittleBeats™ data among children under five years of age in the home environment and validated algorithms (i.e., analytic validation) of infant/child and parent vocalizations using audio data from the LittleBeats™ device [43] and infant/child sleep using all three sensor modalities [44]. Further, we have assessed parents’ perceptions and experiences of using the LittleBeats™ device (i.e., usability testing) with their children in the home across multiple daylong recordings [45]. The current paper complements these prior reports by providing a detailed description of the LittleBeats™ device used for data acquisition, followed by the technical validation of each of the three sensors (ECG, IMU, audio) using standardized laboratory procedures. Finally, although we use signal processing and machine learning algorithms in our technical validation, this work does not constitute an analytic validation because the analytic methods we employ here are well established and have been previously validated. 

### 1.3. Validation of ECG (Study 1, 2), IMU (Study 3), and Audio (Study 4, 5) Sensors

Our first objective is to validate data obtained from the LittleBeats™ ECG sensor. Such data are a critical aspect of the LittleBeats™ platform because they provide key information about physiological stress. Specifically, we aim to assess cardiac vagal tone, which is an indicator of parasympathetic activity reflective of the myelinated vagus (or X^th^ cranial nerve) that provides motor and sensory linkages between the brainstem and visceral organs (e.g., heart and bronchi) [29,46,47]. At rest, higher vagal tone indicates greater regulatory capacity. Under challenge conditions, the withdrawal of the vagal “brake” (and corresponding vagal suppression) supports vigilance of the environment and mobilization in response to the challenge. Respiratory sinus arrhythmia (RSA), a measure of heart rate variability as a function of the rate of spontaneous respiration, is a well established and noninvasive method for assessing cardiac vagal tone in both adults [29,48] and children [49,50,51]. RSA is computed from interbeat intervals (IBI) and is most accurately assessed via ECG. Thus, an ECG sensor is an integral part of the LittleBeats™ platform and enables the assessment of dynamic changes in cardiac vagal tone and reactivity (via RSA) in response to stressors. We recruited an adult sample (Study 1) and an infant sample (Study 2) to assess the performance of the LittleBeats™ 3-lead ECG sensor against the BIOPAC MP160 system (BIOPAC Systems, Inc., Camino Goleta, CA, USA), a gold-standard wireless system for measuring ECG in laboratory studies and one that has been used in similar validation studies [52,53,54]. Because a host of prior studies have validated a laboratory task (i.e., Still Face Paradigm [39,40,41]) and algorithm (i.e., Porges–Bohrer [41,42]) to assess infant stress physiology, we conducted a second laboratory validation of the LittleBeats™ ECG sensor with a small infant sample (Study 2). 

Our second objective was to validate the performance of the LittleBeats’ IMU (Study 3), which integrates an off-the-shelf IMU sensor that has been used in prior studies with excellent performance [55,56,57]. Including an IMU on the LittleBeats™ platform provides information on posture and movements that can be used to assess such constructs as infant physical activity, sedentary behavior, and sleep. For the purpose of this report, we conducted an initial technical validation of the IMU with adults using controlled protocols and key physical activities that have been examined extensively in the literature [58,59,60]. Similar to prior validation studies of IMUs [61,62,63], we conducted an on-body validation experiment on predefined motions collected in the laboratory. Additionally, given the pervasive and effective use of smartphones in IMU-based human activity detection research [58,59,60], we used a smartphone IMU (Google Pixel 1, with the SensorLogic app) as the industry standard [58].

Our third and final objective was to verify the audio quality of LittleBeats™. To this end, we evaluated adults’ speech on two standard speech-processing tasks: speech emotion recognition (SER, Study 4) and automatic speech recognition (ASR, Study 5). In previous studies, algorithm workflows of SER [64,65,66] and infant/parent vocalization classification tasks [67,68,69,70] have shared many similarities, such as (1) extracting paralinguistic or hand-crafted features at the utterance-level and/or acoustic features at the frame-level as input and (2) performing classification tasks using traditional classifiers (e.g., support vector machine and k-nearest neighbor) or neural-network-based models (e.g., convolutional neural networks and recurrent neural networks). These similarities make a technical validation in the context of SER particularly relevant for LittleBeats™ intended use to assess infant vocalizations. Further, although we do not intend to use LittleBeats™ data to transcribe speech recorded in the home, testing LittleBeats™ audio on an ASR task serves as an especially effective indicator of LittleBeats™ audio quality given the established advanced ASR technology [71,72,73] that is capable of dealing with a variety of accents, dialects, and noisy environments. If LittleBeats™ performs well with such advanced ASR systems, it indicates that its audio quality is likely to be very high.

## 2. Overview of LittleBeats™ Platform

**Figure 1:**
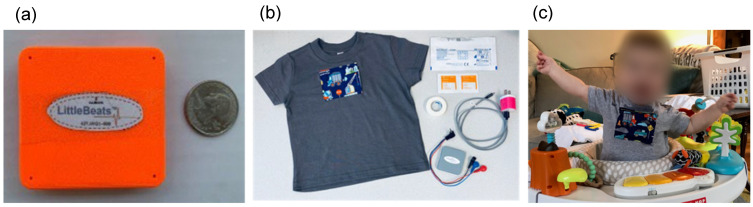
(**a**) LittleBeats™ device case, (**b**) LittleBeats™ supplies including ECG leads, electrodes, charger, and shirt, and (**c**) infant wearing LittleBeats™ in the home. This figure has been previously published [45] under the Creative Commons Attribution License.

To collect synchronized multimodal sensor data suitable for infants and young children, we designed a unique sensing platform called LittleBeats™. All electronics are housed in a 3D-printed case (55 × 57 × 13 mm; see Figure 1), and the device weighs 1.48 ounces (42 g), making it suitable as a child wearable. The LittleBeats™ device is placed in a specially designed t-shirt that the child wears (see Figure 1). A chest pocket with a side opening is centered on the shirt and snugly holds the LittleBeats™ device in place. The inside of the pocket is padded with a thin foam layer for comfort, and two ¼ inch metal snaps are used to securely close the pocket. 

### 2.1. Hardware Design

**Figure 2:**
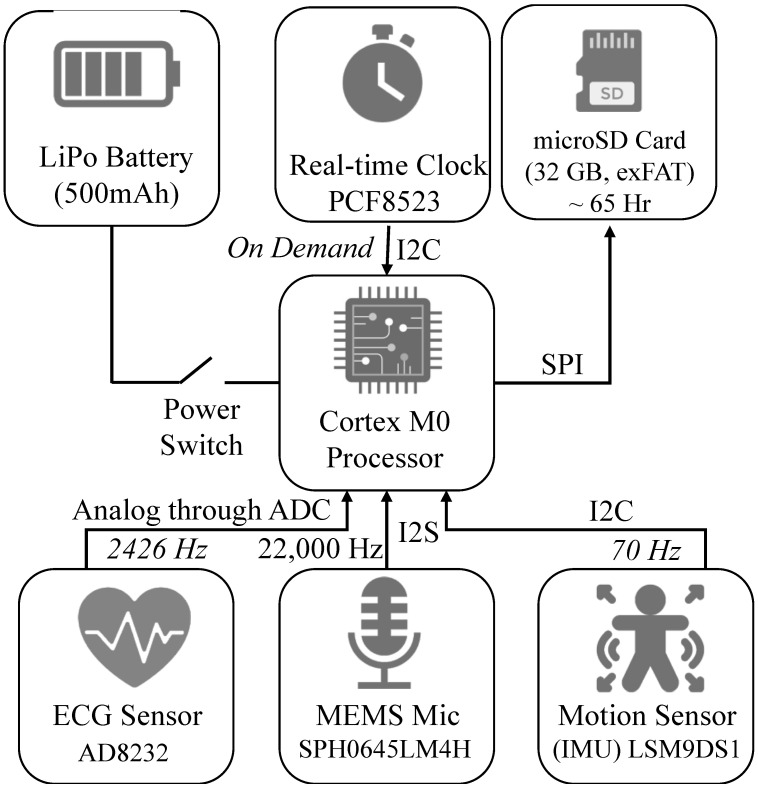
Block diagram of the LittleBeats™ device, including the data-reading protocols. LiPo = lithium polymer, exFAT = extensible file allocation table, ADC = analog-to-digital converter, SPI = serial peripheral interface, ECG = electrocardiogram, MEMS = micro-electromechanical system, IMU = inertial measurement unit.

LittleBeats™ consists of five components: a processing unit, memory unit, time-keeping unit, power unit, and sensing unit (see Figure 2). The technical specifications for each of these components are provided below. LittleBeats™ is not a commercial device, which eliminates concerns about “expiration dates” and permits easy modifications to firmware (e.g., “turning off” one or more of the sensors) to suit research goals. 

#### 2.1.1. Processing Unit

LittleBeats™ is controlled by an ARM Cortex M0 processor, clocked at 48 MHz, and has 256 KB of flash memory and 32 KB of RAM. This processor supports data reading and writing using a serial peripheral interface (SPI), inter-integrated circuit interface (I2C), inter-IC sound (I2S), and an analog-to-digital Converter (ADC). This processor is responsible for collecting data from the sensors, storing the data on the microSD card, and pooling time from the real-time clock using the I2C and SPI. The unit can communicate with Bluetooth Low Energy (BLE), which we currently turn off to reduce energy consumption.

#### 2.1.2. Memory Unit

Besides the flash memory in the microcontroller, to store the collected data, we use a 32 GB microSD card. The microSD socket connects to the microcontroller’s SPI port pins, and the SD card uses exFAT format to maximize the read–write speed. With a 32 GB SD card, we can record up to a total of 65 h of audio, motion, and ECG data across multiple recordings.

#### 2.1.3. Time-Keeping Unit

Though the processing unit has an internal clock, it resets on every reboot of the device and is not synched with the outside world, a functionality that is essential for longitudinal data collection. We use a battery-backed real-time clock (RTC), PCF8523 with 32 kHz crystal, that is interfaced to the microcontroller using the I2C protocol. Note that the processor clock and the RTC are two different clocks; the first is a relative clock that starts from 0 when the system powers on, and the second is a real-time clock with a backup battery and is synchronized with the universal clock, which provides log data timestamps. We have performed a detailed system test and found that this RTC has a drift of 1–3 s every 11–12 h. As our main intention with the RTC is to synchronize the three sensing modalities, this drift does not influence our goal and, thus, does not require regular resynchronization. When preparing the LittleBeats™ device in the lab to send to a new family, the clock is synchronized via the UART interface via the Coordinated Universal Time or UTC clock.

#### 2.1.4. Power Unit

The system is powered by a 500 mAh LiPo rechargeable battery (LP303450) that provides approximately 11 h of operational capability per charge. This battery comes with a Protection Circuit Module and meets national (UL2054) and international (IEC 62133) safety standards, including RoHS compliance. The system is powered on and off by a manual switch for easy usage.

#### 2.1.5. Sensing Unit

LittleBeats™ consists of three different sensor modalities: a microphone, a 3-lead ECG sensor, and an IMU. To record audio, we use a SPH0645LM4H breakout board, which includes a single MEMS microphone and the necessary circuitry to output digital signals (24-bit data) using the I2S protocol. The microphone has a low current requirement of 600 μA and a high signal-to-noise ratio (SNR) of 65 dB. To record ECG, we use the AD8232 heart rate monitor, which measures the electrical activity of the heart and outputs an ECG as an analog reading. Disposable electrodes are connected to the lead wires (20 cm) via button snaps (1 cm), and the three leads are connected to the device via a 2.5 mm jack. To record motion, we use LSM9DS1, which is a 9-degrees of freedom IMU consisting of a 3-axis accelerometer, a 3-axis magnetometer, and a 3-axis gyroscope. These sensors together provide data on acceleration, direction, and orientation, respectively. 

### 2.2. Data Acquisition

We have developed custom firmware for the system written in the C programming language and enable timestamped data streams from all three sensor modalities to be stored on the SD card. For both adult and infant data (including daylong home recordings not reported here), we sample audio at 22 kHz (downsampled to 16 kHz during preprocessing), ECG at 2426 Hz, and 9-axis motion data at 70 Hz. The writing of audio data to the SD card occurs every 10 s, whereas writing the ECG and IMU data occurs every 30 s. These “chunk” durations were determined, keeping the maximum data transfer rate of the peripheral bus of the processor (which is a 32-bit multicentral/multiperipheral bus) in mind. 

The time from the RTC is recorded at the start and end of each data chunk for synchronizing the multiple data streams. We store these data in little-endian binary format unreadable to humans without further processing. These binary files are converted to a human-readable format (.csv for ECG and IMU; .wav for audio) with our custom Python scripts after removing the SD card from the device. The data extraction codes also perform several preprocessing steps (described in the relevant study sections below) to verify and maintain the quality of the data. 

### 2.3. Data Synchronization

Using the time-keeping unit, we synchronize the data collected from three modalities. As these files are written to the SD card in an asynchronized manner, and the sampling rate of each modality is different, there is a need for synchronization. As mentioned in the previous section, each file (or data chunk) in the SD card is timestamped with start and end times. We split the recorded samples into frames (files) by aligning the starting index to the timestamp. The split sample frames are naturally synchronized because the UTC timestamps are consistent across the three sensor modalities. Depending on the version of the device firmware used during data collection, we zero padded the split sample frames for ECG and audio data prior to synchronization to match the expected frame period. IMU data, which are collected at a much lower sampling rate, were not affected by missing samples. 

Importantly, the data collection for the various studies described in this report was slowed due to the COVID-19 pandemic, and in the interim, the device firmware was updated. Studies 1, 3, and 4 reported below used Version 1 firmware, whereas Studies 2 and 5 used Version 2. The key update to Version 2 was switching from a FAT32 (write speed: 108.42 KB/s) formatting of the SDcard to an exFAT (497.33 KB/s) format and the corresponding SdFat Arduino library, which resulted in faster write times and, thus, substantially fewer missing samples in the audio (see Study 4 versus 5) and no missing samples in ECG (see Study 1 versus 2). When applicable, we note the amount of zero padding (i.e., missing samples) for the sensor modality under investigation.

## 3. Study 1: Validation of ECG Sensor–Adult Sample

### 3.1. Materials and Methods

#### 3.1.1. Participants

Sixteen adult participants (56.3% female; mean age = 27.4 years, *SD* = 8.82, range: 18–46) were recruited through a university listserv and flyers displaying study information posted in multiple university buildings. Both forms of recruitment reached adults across various educational and racial/ethnic backgrounds. Participants reported on their highest level of education (13% high school graduate, 20% some college, 40% bachelor’s degree, 27% advanced degree) and their race and ethnicity (33.3% Asian, 60% White non-Hispanic, 6.7% Hispanic). Participants were eligible to participate if they met the following criteria: (a) at least 18 years of age and (b) no known heart problems or abnormalities. 

#### 3.1.2. Study Procedure

Participants visited the laboratory and were guided through a series of tasks while wearing two ECG monitors: (a) LittleBeats™ (Version 1 firmware) and (b) the BIOPAC MP160 system (BIOPAC Systems, Santa Barbara, CA, USA). Six disposable, pregelled, signal-conditioning electrodes were placed on the participant (3 electrodes per device): two below the left clavicle, two below the right clavicle, and two just below the ribcage (i.e., Einthoven’s triangle). Pairs of electrodes were placed side by side but did not touch or overlap. The LittleBeats™ device and BIOPAC BioNomadix wireless transmitter were placed in a specially designed t-shirt with two chest pockets, providing a form factor that was comparable across the two devices and mirrors the form factor used with infant and child participants. BIOPAC samples ECG at 1000 Hz.

Participants were video recorded while completing the following tasks: (a) a 3 min baseline, which involved viewing a clip from a calming video of sea animals, (b) a 4 min puzzle task, which involved solving a 14-piece Tangram puzzle, (c) a 2 min recovery using another clip from the video viewed during the baseline session, and (d) a 4 min nonverbal abstract reasoning task using Raven’s Progressive Matrices (standard version) [74]. The puzzle and matrices tasks each presented a cognitive challenge, and such tasks have been used successfully in prior research to elicit a physiological stress response (i.e., cardiac vagal withdrawal) among adults [75,76,77] and children [78,79] alike. Further, participants completed the two challenge tasks (i.e., puzzle and matrices) while a large countdown timer was displayed on the computer screen, thereby increasing potential stress. For the Tangram puzzle task, eight participants completed the puzzle in under 4 min (*M* = 2.63, *SD* = 0.87), and the ECG data for these participants included only the time in which the participant was engaged in solving the puzzle. The Raven’s Progressive Matrices include sixty multiple choice items; items are organized within five sets (twelve items each), and items within each set increase in difficulty. Participants were instructed to complete as many items as possible within the time allotted, and as expected, no participants completed all items within the 4 min timeframe (*M* items completed = 28.31, *SD* = 6.22).

#### 3.1.3. Data Processing

We implemented the following data pre/postprocessing steps to extract IBI values from the ECG LittleBeats™ and BIOPAC data and compute RSA values: (1) CardioPeak & Segmenter for LittleBeats™ v1.0 [80] was used to extract the R-R peaks from the LittleBeats™ and BIOPAC ECG data and derive the time in milliseconds between consecutive R peaks (i.e., IBI values, 250 Hz sampling rate). This software outputs separate IBI files for each task/session (task time information, which is derived for BIOPAC and LittleBeats™ from the video and audio recordings, respectively, provided in a separate CSV file serves as an additional input file). (2) To correct for artifacts due to zero padding (*M* = 2.36% missing samples, *SD* = 0.14%) in Version 1 of the device firmware, we passed the IBI data through a custom filtering script that took into account missing data samples and used standard IBI artifact detection and editing approaches [81] to correct IBI points due to missing samples. (3) LittleBeats™ and BIOPAC IBI for each task were manually aligned in time by plotting IBI values from each device as a function of time in Excel (see Supplementary Materials for example plots for Study 1 adult data [Figure S1]). (4) All IBI data files were reviewed and, when needed, manually edited using CardioEdit v1.5 by members of our research team who had been previously trained and certified by the Porges’ Brain-Body Center for Psychophysiology and Bioengineering (BBCPB) at the University of North at Carolina Chapel Hill. (5) RSA was computed from BIOPAC and LittleBeats™ IBI data using the Porges–Bohrer algorithm [42] by calculating the natural logarithm of the variance of heart period within the frequency bandpass related to respiration (0.12−0.40 Hz for adults) in CardioBatch Plus [82] software. Within each task, RSA values were computed in 30 sec epochs and then averaged across epochs to obtain task-level means. 

Data from an additional seven participants were collected but were excluded because for one or more of the target sessions (baseline, puzzle, recovery, matrices), the BIOPAC file could not be edited due to an extreme value and/or more than 5% edits (*n* = 4), technical problems with the video recording, which was needed to align the two files at the session level (*n* = 2), and fewer than 90 s of data available (*n* = 1).

### 3.2. Results

We present three sets of analyses. First, we computed error statistics in the LittleBeats™ IBI values via (a) mean error (i.e., average difference between BIOPAC and LittleBeats™ IBI values), (b) mean absolute error (i.e., average absolute difference between BIOPAC and LittleBeats™ IBI values), and (c) mean absolute percent error (i.e., MAPE; mean of absolute error divided by BIOPAC IBI value and multiplied by 100). MAPE is a widely used metric in the validation of physiological sensors, and an error rate of ±10% has been deemed acceptable for ECG-related measurements in recent studies [83,84,85] and by the Consumer Technology Association [86]. The number of total IBI data points and error statistics for each task are shown in Table 1.

The MAPE was under 6% for all tasks across all participants. MAPE values were also computed separately by participant and ranged from 0.57% to 13.64% for the baseline, 0.59% to 11.74% for the puzzle task, 0.57% to 11.31% for recovery, and 0.63% to 12.39% for the matrices task. Of the 64 MAPE scores (16 participants × 4 tasks), 26 were under 5%, 33 were under 10%, and 5 were between 10% and 13.64% percent. Data from the same participant yielded the lowest MAPE values across all tasks, whereas data from two participants yielded the highest MAPE values (baseline and matrices for one participant; puzzle and recovery for the other). For descriptive purposes, we computed the bivariate correlational value between BIOPAC average IBI values and MAPE scores. Weak-to-moderate positive associations emerged, although associations were not statistically significant (*r*s = 0.24 0.45, 0.26, 0.21, *p*s = 0.37, 0.08, 0.33, 0.44, baseline, puzzle, recovery, and matrices tasks, respectively). Scatterplots of these associations indicated a positive association between BIOPAC IBI average scores and MAPE until IBI scores reached approximately 0.90 s; the few cases with an average IBI score greater than 0.90 s showed no discernible increase in MAPE.

**Figure 3:**
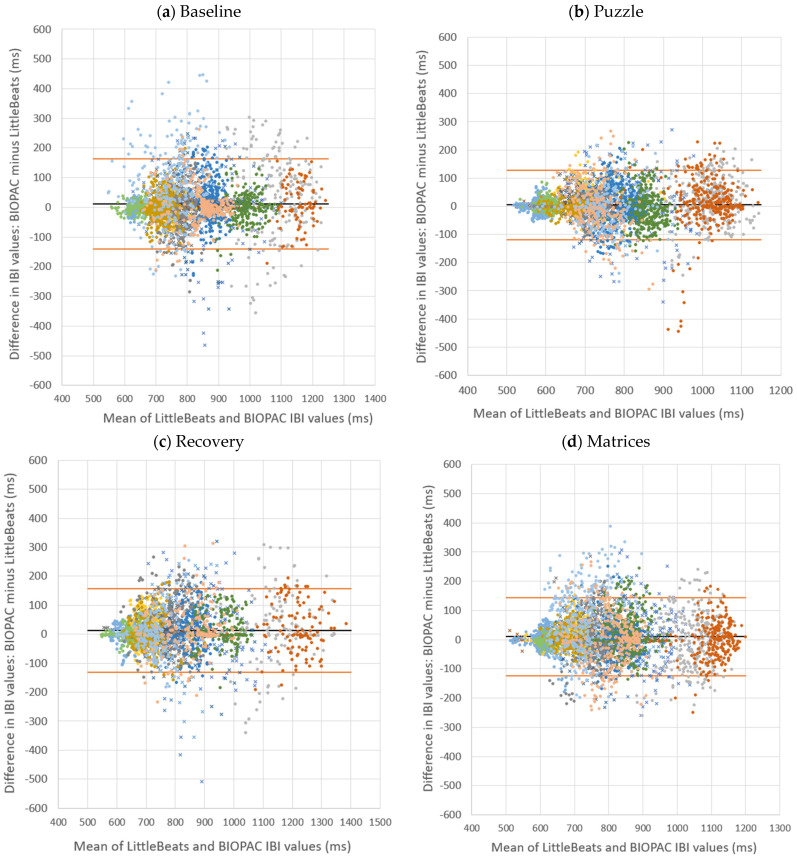
Bland–Altman plots comparing interbeat intervals (IBI) extracted from LittleBeats™ and BIOPAC ECG signals for four laboratory tasks completed by adult participants (*N* = 16, Study 1). Each color-shaped symbol represents IBI data from a specific participant. ms = milliseconds.

Second, Bland–Altman plots provide a direct and appropriate comparison between quantitative measurements of the same phenomenon [87]. Bland–Altman plots of IBI values, in which the X axis represents the mean of the two instruments (LittleBeats™, BIOPAC) and the Y axis represents the difference (in milliseconds) between the two instruments (BIOPAC minus LittleBeats), are shown in Figure 3. IBI values are plotted separately by task and color coded by participant. Bland–Altman plots can be used to assess the presence of outliers (with respect to differences in the two measurements) or whether data are systematically biased (i.e., difference between measures is consistently in one direction). Across tasks, the mean error (BIOPAC–LittleBeats™) in IBI values ranged from 4.5 milliseconds (puzzle task) to 12.7 milliseconds (recovery) as shown in Table 1 above, indicating the BIOPAC and LittleBeats™ IBI values were typically within hundredths or thousandths of a second and that, on average, LittleBeats™ (vs. BIOPAC) IBI values were slightly lower. The Bland–Altman plots also show that 95% of the BIOPAC–LittleBeats™ errors (difference scores) fall within a range of approximately± of 150 milliseconds (see Table 1 for specific 95% limits of agreement for each task). Further, errors are smaller at the lower end of observed IBI values (i.e., ~500 to ~700 milliseconds on the X axis) and are more dispersed at the middle and higher ends of observed IBIs (i.e., ~800 to ~1200 milliseconds), although this pattern varies as a function of case and task (e.g., the case shown in peach exhibits moderate levels of IBI, with a lower error rate in the baseline task but more dispersion in errors in the puzzle, recovery, and matrices tasks). Finally, errors show a relatively even distribution around the mean error (black line) and limits of agreement (orange lines) in the Bland–Altman plots across tasks and individuals, indicating little systematic bias in the errors.

**Figure 4:**
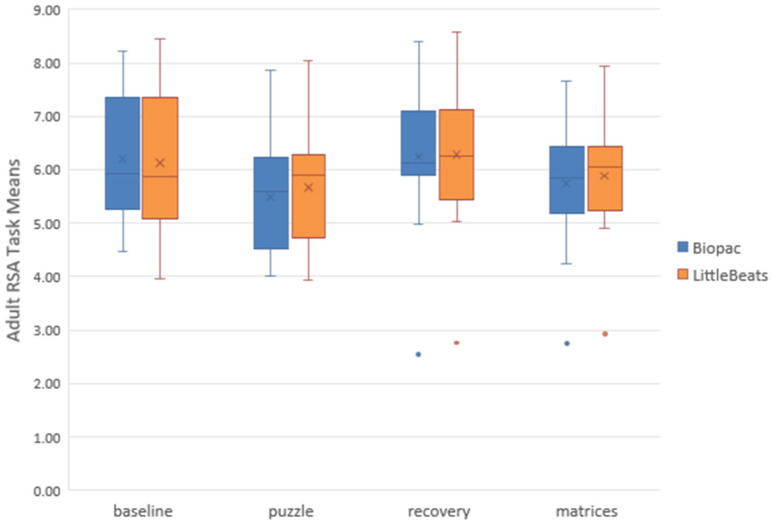
Box and whisker plots depicting BIOPAC and LittleBeats™ respiratory sinus arrhythmia (RSA) values. Task mean indicated by X, median indicated by horizontal line, range indicated by end points of the vertical lines or “whiskers,” and outliers indicated by dots.

Our third and final analysis focused on RSA measurements derived from the IBI data (see Data Processing section above). We plotted the RSA sample means and distributions for each task (see Figure 4). Because the puzzle and matrices tasks each presented a mild to moderate challenge, we expected RSA to decrease from baseline to the puzzle task, increase from puzzle to recovery, and decrease again from recovery to the matrices task. Paired *t*-tests indicated significant (*p* < 0.05, one-tailed) and hypothesized differences in RSA means across tasks: (a) baseline minus puzzle, *t*(15) = 2.71 and 1.78, *p* = 0.008 and 0.047, BIOPAC and LittleBeats™, respectively, (b) puzzle minus recovery, *t*(15) = −2.30 and −1.96, *p* = 0.018 and 0.034, and (c) recovery minus matrices, *t*(15) = 2.36 and 2.00, *p* = 0.016 and 0.031. Thus, despite a degree of error in the LittleBeats™ IBI values, expected task-related changes in RSA were observed and mirrored RSA changes assessed via IBI data obtained from the BIOPAC system. 

## 4. Study 2: Validation of ECG Sensor–Infant Sample

### 4.1. Materials and Methods

#### 4.1.1. Participants

We recruited five infants (3 females, M_age_ = 7.64 months, age range: 4–12 months) via an announcement posted on a university-wide listserv. Paralleling Study 1 procedures, infant ECG data were collected simultaneously by LittleBeats™ and the BIOPAC MP 160 system in the laboratory. Due to the burden of wearing two ECG monitors simultaneously and because results from Study 1 indicated an acceptable agreement between the two devices, we limited our infant sample to five participants across a wide range of ages during the first year of life. Infants were eligible to participate if they met the following criteria: (a) under 12 months of age, (b) no known cardiac abnormalities, and (c) their mother was willing to speak English during the visit if English was not her native language. All ECG data collected are included in the analyses below. 

#### 4.1.2. Study Procedure

Infant–mother dyads participated in a laboratory visit, in which infants wore the LittleBeats™ (Version 2 firmware) and BIOPAC ECG sensors (BIOPAC Systems, Santa Barbara, CA, USA). The LittleBeats™ device and BioNomadix wireless transmitter were placed in dual chest pockets of a specially designed infant shirt. While seated on their mother’s lap, infants were video recorded during a 3 min baseline session that was identical to the baseline video session used in Study 1. Following the baseline session, infants and mothers were observed in the Still Face Paradigm (SFP) [39], which consisted of three 2 min episodes: (1) play, while infant was seated in a bouncy seat or high chair (depending on age), (2) still face, in which mothers were cued (via a brief knock on the playroom door) to cease verbal and physical interaction with their infant while looking at the infant with a neutral face, and (3) reunion, in which mothers were cued (via a brief knock) to resume interacting with their infant. No toys were present during the SFP, and mothers were asked to not take their infant out of the seat. The still face episode of the SFP is emotionally challenging for infants and typically elicits a distress response [41]. If the infant displayed high levels of prolonged distress (i.e., 15–20 s) during the still face episode, the episode was curtailed. The mother–infant interaction during the SFP was video recorded via two remote-controlled cameras with pan/tilt/zoom features; the cameras were mounted on opposite corners of the playroom and controlled from an adjacent observational booth.

#### 4.1.3. Data Processing

Processing of the BIOPAC and LittleBeats™ ECG, IBI, and RSA data were identical to the steps outlined in Study 1 with the following exceptions. First, Version 2 of the device firmware results in no missing ECG samples and, thus, we did not implement the custom filtering script that automated the correction of IBI points due to the missing samples described in Study 1 (Data Processing; Step 2). Second, in computing RSA values for the infant data, the natural logarithm of the variance of heart period within the frequency bandpass related to respiration for infants (i.e., 0.3–1.3 Hz) [88] was calculated in CardioBatch Plus [82] software. See Figure S2 in the Supplementary Materials for example plots of aligned LittleBeats™ and BIOPAC IBI values for an infant participant.

### 4.2. Results

We present four sets of analyses. First, we computed the same error statistics reported in Study 1 (i.e., mean error, mean absolute error, MAPE). As shown in Table 2, the MAPE was under 2% for all tasks across all participants. Within participants, MAPE ranged from 0.86% to 1.54% for the baseline, 0.74% to 1.10% for the SFP play episode, 0.82% to 3.65% for the SFP still episode, and 0.69% to 2.23% for the SFP reunion episode. Of the 20 MAPE scores (5 participants × 4 tasks), 9 were under 1%, 9 were under 2%, and 2 scores were 2.23% and 3.65%, respectively. 

**Figure 5:**
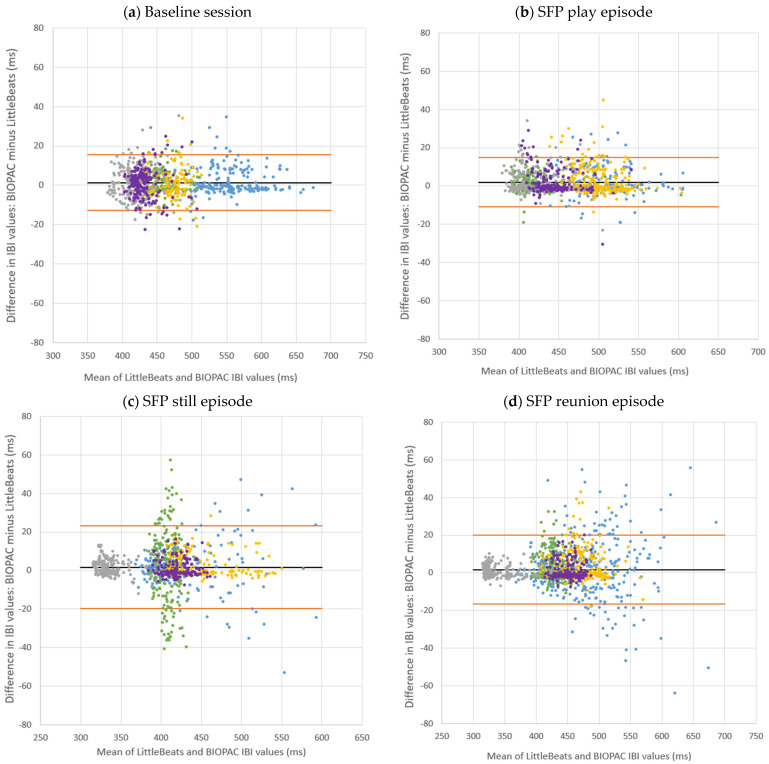
Bland–Altman plots comparing interbeat intervals extracted from LittleBeats™ and BIOPAC ECG signals for the baseline session and episodes of the Still Face Procedure (SFP) for infant participants (*N* = 5, Study 2). Each color represents the IBI data from a specific participant. ms = milliseconds.

Next, Bland–Altman plots (by task and color coded by participants) are shown in Figure 5, with the corresponding statistics reported in Table 2. The mean error (BIOPAC–LittleBeats) in IBI values ranged from 1.3 milliseconds (baseline) to 2.0 milliseconds (SFP play), indicating the BIOPAC and LittleBeats™ IBI values were typically within thousandths of a second and that, on average, LittleBeats™ values were slightly lower than BIOPAC values. The Bland–Altman plots also show that 95% of the BIOPAC–LittleBeats™ errors (difference scores) fall within an approximate range of ±15 to 20 milliseconds (see Table 2 and Figure 5 for specific 95% limits of agreement for each task). Further, for the baseline and SFP play episode, errors are consistently small and approach zero across the range of observed IBI values (~375–675 milliseconds on the X axis). For the SFP still and reunion episodes, the errors, although still relatively small, are more dispersed, particularly at the higher end of the observed IBI values (i.e., ~450 to ~650 milliseconds). Finally, across tasks and individuals, the errors are distributed relatively evenly around the mean error (black line) and limits of agreement (orange lines), suggesting little systematic bias in the ECG/IBI data derived from the LittleBeats™ device. 

**Figure 6:**
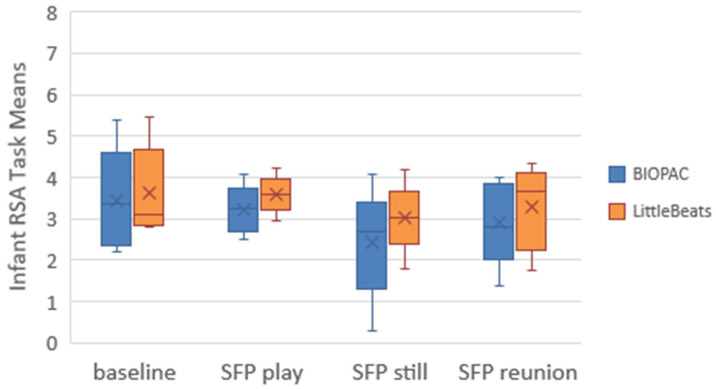
Box and whisker plots depicting BIOPAC and LittleBeats™ respiratory sinus arrhythmia (RSA) values. Task mean indicated by X, median indicated by horizontal line, and range indicated by end points of the vertical lines or “whiskers”. No outliers were observed.

Third, we examined the RSA task mean to assess the pattern of change across the baseline and SFP episodes, although the small sample size prohibited statistical tests. Based on a host of prior studies (see [41] for a meta-analytic review), we expected to observe the highest RSA values during the baseline and SFP play sessions (indicative of low-stress contexts) and the lowest RSA values (indicative of an RSA withdrawal in response to a stressor) during the SFP still episode, with modest increases in RSA during the reunion episode, indicating a partial recovery from the stress of the SFP still episode. We plotted the RSA sample means and distributions for each task (see Figure 6). Although RSA values based on the LittleBeats™ data are consistently higher than values from the BIOPAC data, the more important finding is that LittleBeats™ data followed the same task-related changes in RSA observed in the BIOPAC data, indicating sensitivity to within-person changes in RSA.

## 5. Study 3: Validation of Motion Sensor–Activity Recognition

### 5.1. Materials and Methods

#### 5.1.1. Participants

Twelve adults (66.7% female; mean age = 24.7 years, *SD* = 5.42, range: 18–33) were recruited through online announcements at a university in a mid-sized midwestern city. Participants reported on their highest level of education (16.7% high school graduate, 25% some college, 41.7% bachelor’s degree, 16.7% advanced degree) and their race and ethnicity (66.7% Asian, 33.3% White, non-Hispanic). 

#### 5.1.2. Study Procedure

Participants wore the LittleBeats™ (Version 1 firmware) and the smartphone in two chest pockets of a custom t-shirt, and the smartphone and LittleBeats™ device each fit snugly in their respective shirt pocket, permitting a comparable form factor (note that other more expensive and precise IMUs (e.g., Xsens [89]) that are worn with form-fitting chest straps do not permit a parallel form factor). Participants were video recorded while performing a series of six physical activities (i.e., sit, stand, walk, glide or walk sideways, squat or deep knee bends, and rotating in chair) commonly used in the activity recognition literature [58,59,60]. Here, sitting and standing captured the stability of the data, while walking, gliding, and squatting captured acceleration along the three different axes of the accelerometer. Rotation captures the performance of the gyroscope. The following are the six task descriptions:

1. The participant sits on a chair and watches a video for 2 min.
2. Between each activity, the participant stands for 30 s.
3. The participant walks to the end of the room and back three times.
4. The participant glides or steps to the left until they reach the end of the room, then glides or steps to the right until they reach the other end of the room, for one minute.
5. The participant completes squats or deep knee bends for one minute.
6. The participant sits in an office chair and rotates slowly five times.

#### 5.1.3. Data Processing

**Figure 7:**
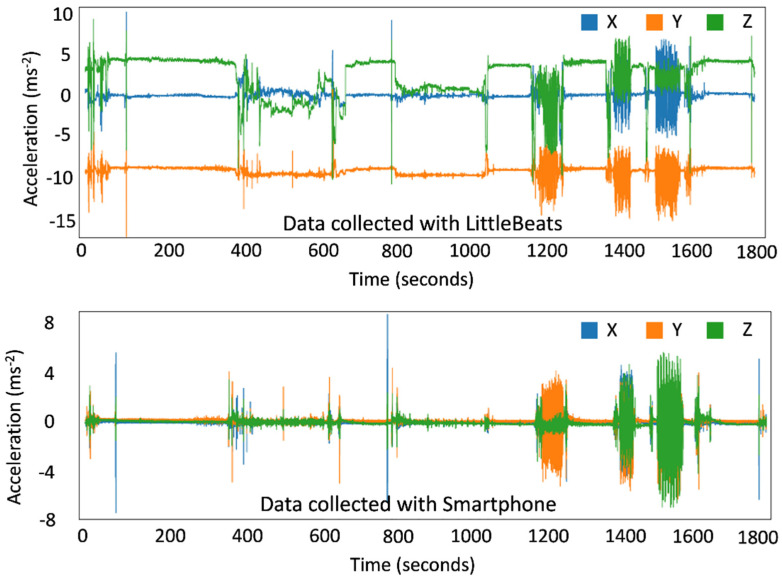
A sample of raw accelerometer data in 3D space collected with LittleBeats™ (**top panel**) and a smartphone (**bottom panel**) while performing motion activities. XYZ represent the Cartesian coordinates of 3-dimensional space; X (shown in blue) = motion along the horizontal axis (side to side), Y (shown in orange) = motion along the vertical axis (up and down), Z (shown in green) = rotation or forward movement.

The smartphone uses an off-the-shelf IMU data collection app named “SensorLogic” (SensorLogic, Bozeman, MT, USA) that collects the data and provides processed accelerometer data mitigating the effect of gravity and noise on the IMU, as shown in Figure 7. The microcontroller on the LittleBeats™ device reads the IMU data directly from the appropriate registers with a function call. We remove the gravitational effect from the LittleBeats™ data by subtracting the gravitational acceleration (9.8 m/s) from the affected axis of the accelerometer data. Note that the smartphone performs this action internally. 

Due to the asynchronous collection of the IMU data with the ADC, the sampling rate of the IMU data collection is dynamic with an offset of ±5 Hz. Because we use traditional off-the-shelf algorithms to validate the IMU sensor data and because such traditional machine learning and signal processing algorithms take input with a fixed sampling rate, the dynamic sampling rate of LittleBeats™ requires correction. To this end, we utilize the timestamp from the time-keeping unit and use a sliding window to determine the number of samples in each nonoverlapping 30 s interval. We then upsample (with interpolation) or downsample the 30 s based on whether we have more or fewer data points than the required sampling rate.

### 5.2. Results

#### 5.2.1. Data Distribution and Balancing

Among the six tasks, five were relevant to assessing the performance of the accelerometer: sit, stand, walk, glide, and squat. Because the accelerometer on the chest fails to differentiate between sitting and standing still, we combined these two activities under a single label (“upright”). We take 5 s segments and label each segment with the activity label, which yields a total 1254 samples across all four activities with the following distribution: 812 upright, 150 walk, 176 glide, and 116 squat. Note that we have an imbalanced dataset where all classes do not have the same number of samples, and we omit samples where the participant transitions from one activity to another. 

We randomly split the data into training and test sets with 80% training and 20% testing samples, while ensuring that samples from all classes are present in both the training and testing datasets. Using 10-fold cross-validation, we eliminate any bias of the train–test split. We normalize the data by removing the mean and scaling to unit variance. We use these normalized samples as the input to the classifier.

#### 5.2.2. Classification

We classify each 5 s segment using a multiclass random forest classifier for the following four-way classification problem: upright vs. walk vs. glide vs. squat. Random forest is a meta-estimation technique that fits a number of decision trees on multiple subsamples of the dataset and then takes the average. This averaging increases the prediction accuracy and controls for overfitting. We use 100 decision trees in our random forest and use entropy to measure the quality of a split. 

We report the mean and standard deviation of three metrics across ten data splits to evaluate classification performance. These metrics are (1) accuracy, which captures the overall level of agreement between the classifier and the ground truth, (2) F1-score, which represents the harmonic mean of precision and recall, where precision (or “positive predictive value”) is the number of true positive predictions divided by the number of all positive predictions and recall (or “sensitivity”) is the number of true positive predictions divided by the number of all true positives, and (3) Cohen’s kappa [90]. Chance (a classifier that assigns labels uniformly at random) would achieve an accuracy of 25%, an F-1 score slightly below 25% (because of class imbalance), and a kappa value of 0.0. Kappa values between 0.60 to 0.80 indicate moderate agreement and are considered acceptable; kappa values greater than 0.80 indicate substantial agreement and are considered excellent [91].

**Figure 8:**
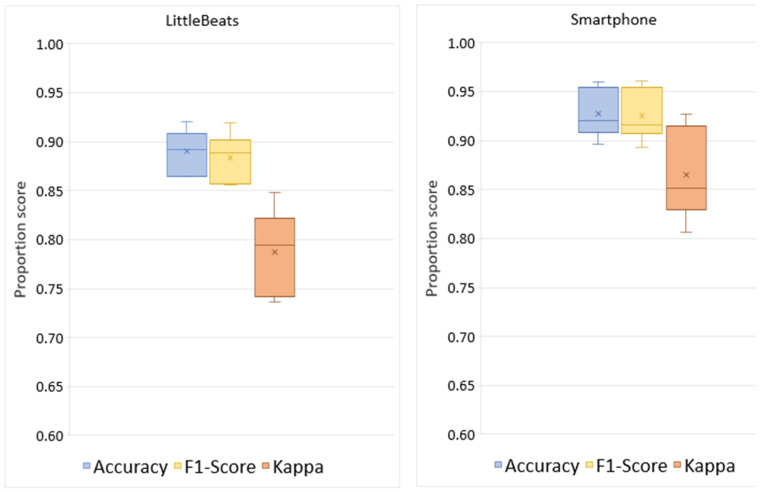
Box and whisker plots depicting the performance metrics (accuracy, F1-score, kappa) of activity classification (4 classes: upright, walk, glide, squat) with LittleBeats™ and smartphone IMU data (Study 3). The mean proportion score on a given performance metric is indicated by X, the median is indicated by a horizontal line, and the range is indicated by the end points of the vertical lines or “whiskers”. No outliers were observed.

Table 3 shows the confusion matrices for the four-way activity classification separately for the LittleBeats™ and smartphone data, and Figure 8 shows the related performance metrics. Although the algorithm for activity detection performed better when applied to data from the smartphone, the performance metrics on LittleBeats™ data also showed high levels of accuracy (89%), F1-score (88%), and Cohen’s kappa (0.79) and are therefore on the boundary between acceptable and excellent. Further, the F1-score for LittleBeats™ versus smartphone data represents a decrease in performance of less than 4 percentage points. 

To evaluate whether there was a significant difference in overall classification errors using LittleBeats™ versus smartphone data, we conducted a McNemar’s test [92], which is appropriate to use with paired nominal data representing two categories (e.g., correct versus incorrect prediction). A nonsignificant test would indicate that the classification error rates (or conversely, rates of correct classification) do not differ across devices. Computing the McNemar’s test using the 2 × 2 contingency matrix shown in Table 4 yielded a McNemar significant Chi-squared statistic of 7.41, *p* = 0.006, which suggests that performance is significantly different across the two devices, with LittleBeats™ data yielding more errors than the smartphone data. 

Finally, we test the Gyroscope using data from the sixth activity (i.e., rotate in chair). With the Gyroscope data alone and using a rule-based model (decision tree), we were able to classify rotations in the chair with >99% accuracy for data from both the smartphone and LittleBeats™, where we have two classes (i.e., rotation versus all other activities). High levels of accuracy are possible because of the distinct 360 degree rotation at one axis of the Gyroscope in this activity. 

## 6. Study 4: Validation of Audio Sensor—Speech Emotion Recognition

### 6.1. Materials and Methods

#### 6.1.1. Participants

Eight adults (50% female; mean age = 29 years, *SD* = 13.10, range: 18–55), including six undergraduate students who majored in theater (3 males and 3 females) and researchers in our team who had amateur acting experience (1 male and 1 female), participated. Participants reported on their highest level of education (50% some college, 25% bachelor’s degree, 25% advanced degree) and their race and ethnicity (12.5% Black, 62.5% White non-Hispanic, 12.5% Hispanic, 12.5% more than one race).

#### 6.1.2. Study Procedure

We partially replicated the procedures of collecting emotional speech in the Ryerson Audio–Visual Database of Emotional Speech and Song (RAVDESS) [93] corpus in a smaller scale in terms of the number of participants and emotion types. RAVDESS corpus contains the speech of 24 professional actors (12 female, 12 male), vocalizing two lexically matched statements, “Kids are talking by the door” and “Dogs are sitting by the door.” Eight emotional speech samples, including neutral, happy, sad, angry, fearful, surprise, and disgust expressions are recorded. Each expression is produced at two levels of emotional intensity (normal, strong). 

Paralleling our validation of the motion sensor (Study 3), each participant wore a specially designed shirt that held both the LittleBeats™ device (Version 1 firmware) and a smartphone (Google Pixel, 1st generation), and both LittleBeats™ and the smartphone were used to simultaneously record participants’ speech. The smartphone was used as the industry standard and enabled both high-fidelity recordings as well as a comparable form factor. Participants were asked to read the two statements used in the RAVDESS study (“Kids are talking by the door” and “Dogs are sitting by the door”) 1–2 times in a neutral voice and 2–3 times for each of the six emotion types (i.e., happy, sad, angry, fearful, surprised, disgusted), but without varying emotional intensity. 

#### 6.1.3. Cross-Validation Check on Emotional Speech Corpus

To verify the quality of our emotional speech corpus, three human raters labeled each utterance using one of the above six emotion labels. Both LittleBeats™ and smartphone audio clips (one utterance per clip) were randomly shuffled before distributing to the human raters. Because interrater reliability scores fell below 0.60 for clips expressing fear, disgust, and surprise, we limited our validation experiment to 4 classes: neutral, happy, sad, and angry. Most participants read each of the two statements once in a neutral voice and two times for each emotion. To collect more samples of emotional speech, the last three participants read each statement three times for each emotion category, including neutral, resulting in a total possible dataset of 142 utterances for neutral, happy, sad, and angry combined. One happy utterance was excluded from our dataset due to lack of agreement among the three human raters. Thus, our dataset includes 141 samples (neutral: 28, happy: 37, sad: 38, angry: 38) for both LittleBeats™ and the smartphone.

#### 6.1.4. Audio Data Processing

As most state-of-the-art acoustic algorithms and pretrained models use audio at 16 kHz, we downsampled our collected 22 kHz samples to 16 kHz. In instances of high-frequency audio clipping, we further processed the audio stream using the built-in clipfix function of Audacity^®^ software (version 3.3.3 [94]) that finds the clipped regions of LittleBeats™ audio and performs interpolation of the lost signals for declipping. We empirically set the threshold of clipping to 70% without reducing amplitude for restored peaks to obtain superior audio quality. Using Version 1 of the firmware, the average proportion of missing samples, computed as a function of expected samples based on the UTC timestamps, was 0.087 (*SD* = 0.153).

### 6.2. Results

**Figure 9:**
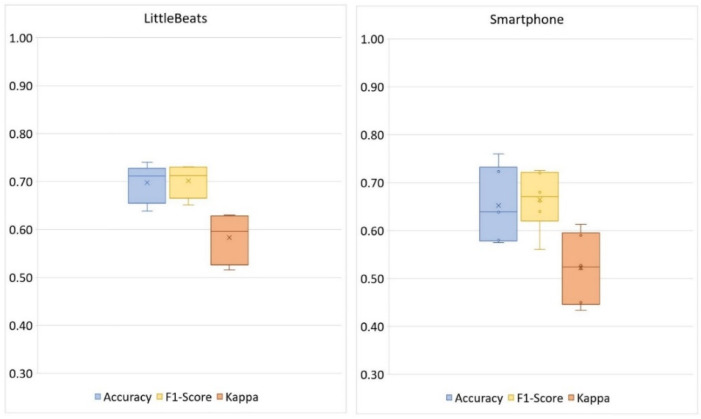
Box plots showing the performance of speech emotion recognition (4 classes: neutral, happy, sad, angry) with LittleBeats™ and smartphone audio data (*N* = 8, Study 4).

Given a relatively small corpus, we used the sklearn package [95] to implement a linear discriminant analysis (LDA) for our SER validation task. We randomly split our corpus into 3 folds and performed 3-fold cross-validation tests. Table 5 shows the confusion matrices for the four-way emotional speech classification separately for LittleBeats™ and smartphone data, and Figure 9 shows the related performance metrics (i.e., accuracy, F1-score, Cohen’s kappa scores). As seen in the figures, performance on speech emotion recognition tended to be higher for data collected with the LittleBeats™ device versus the smartphone.

Next, we conducted a matched-pairs test [92] to assess whether the performance on our speech emotion recognition task differed significantly between the two recording devices (see Table 6 for the 2 × 2 contingency matrix between LittleBeats™ and smartphone data). The test was nonsignificant (*p* = 0.26), indicating the LDA performed equally well when using audio from the LittleBeats™ device versus the smartphone.

## 7. Study 5: Validation of Audio Sensor—Automatic Speech Recognition

### 7.1. Materials and Methods

#### 7.1.1. Participants

Twelve adults (58.3% female; mean age = 21.74 years, *SD*= 3.18, range = 18–26) were recruited via a university listserv and posted flyers. Participants reported on their highest level of education (8.3% high school graduate, 58.3% some college, 8.3% associate’s degree, 25% bachelor’s degree) and their race and ethnicity (16.7% Asian, 16.7 Black, 66.7% White non-Hispanic).

#### 7.1.2. Study Procedure

Paralleling procedures in Studies 3 and 4 above, participants wore LittleBeats™ (Version 2 firmware) and a Google Pixel smartphone in a t-shirt with dual pockets. While seated at a desk, participants read the Rainbow Passage aloud. The Rainbow Passage [96] (330 words), which includes a variety of sounds and mouth movements used in unscripted English speech, has been widely used in prior work to assess speech production and reading fluency [97,98]. 

We used wav2vec 2.0 (W2V2 [71]) as the off-the-shelf software to perform our ASR validation task. W2V2 is a recently published model that uses unsupervised pretraining from ~52k-h of unlabeled raw wav audio, and then excels on multiple speech-processing tasks. For example, W2V2 with Connectionist Temporal Classification (CTC) loss at the character-level achieved a competitive performance on the test–clean set of Librispeech corpus (1.9% word error rate [WER] [71]). 

We implemented W2V2 using the SpeechBrain framework [99]. The W2V2 requires input audio as raw wav files sampled at 16k Hz. For smartphone audio passages, the raw recordings were sampled at 44.1 kHz and stored in .m4a format. We used ffmpeg software (version 4.4.4, [100]) to convert the smartphone recordings from .m4a format to .wav format and downsampled them from 44 kHz to 16 kHz. Identical data processing steps outlined in Study 4 were used for LittleBeats™ audio recordings (e.g., audio was sampled at 22 kHz and downsampled to 16 kHz). Using Version 2 firmware, the average proportion of missing samples was 0.023 (*SD* = 0.007), which represents a significant decrease from the proportion of missing samples in Study 4 (Version 1 firmware).

We prepared ground truth transcripts using the smartphone audio passages. Annotators manually added repeated words or deleted omitted words if a participant did not read the Rainbow Passage verbatim. We pretrained a bigram language model for the Rainbow Passage using KenLM software (https://kheafield.com/code/kenlm/; accessed 26 June 2023 [101]). We performed both CTC greedy decoding and beam search decoding with beam size 25 and set the language model weight to either 0.0 (no language model) or 2.0 (language model included with a large weight). 

### 7.2. Results

Table 7 below shows the WER for LittleBeats™ and smartphone audio with and without the language model. WER is measured by the edit distance between the reference transcripts and hypothesis transcripts generated by the ASR system. WER can be computed using the following formula, $WER=\frac{S+D+I}{N},$ where S, D, and I are the number of substitution errors, deletion errors, and insertion errors, respectively, and *N* is the total number of referenced words. 

Both LittleBeats™ and smartphone audio passages show relatively good baseline WER using CTC greedy decoding. With beam search, error rates for both the LittleBeats™ and smartphone audio increased slightly compared with the model using greedy decoding. This increase in error rate may be due to beam search bias towards the most probable sequence of words in a small corpus, which may not fully capture the underlying acoustic information. With language modeling, LittleBeats™ and smartphone audio passages have large relative WER improvements (27.6% for LittleBeats™ and 23.7% for smartphone). Overall, although smartphone audio has a somewhat better performance, both LittleBeats™ and smartphone audio show strong performance on this open-vocabulary ASR task.

## 8. Discussion

Studying infants and young children in their natural environments without researchers present poses unique challenges. Unlike research with adults, commercially available wearables (e.g., FitBit, Apple watch, chest strap heart rate monitors) are not feasible for use with infants. Our interdisciplinary team has developed a compact, lightweight device that captures key physiological and behavioral signals unobtrusively in the home and without researchers present. In prior reports, we have demonstrated the usability of LittleBeats™ with infants and young children in the home environment [45], as well as the analytic validation of algorithms to detect infant and caregiver vocalizations using LittleBeats™ audio data [43] and sleep/wake states using synchronized LittleBeats™ data from all three sensors [44] among children from 2 months to five years of age during daylong recordings. The current report complements this prior work by presenting a technical validation, in which we compare the performance of each LittleBeats™ sensor against gold-standard devices that have been used extensively in the prior literature and permit a comparable form factor. We also use algorithms that have been established and verified in the literature and compare the performance of these algorithms using LittleBeats and gold-standard sensor data (see Table S1 in Supplementary Materials for a summary of the algorithms and software included in this report). Due to the feasibility issues of conducting technical validations of the IMU and audio data under controlled conditions with infants and young children, we conducted validations with adult participants only for these modalities using controlled laboratory tasks prevalent in prior work. Below, we discuss the findings for each sensor modality. We also present limitations of the current work and directions for future research with the LittleBeats™ platform.

### 8.1. ECG Sensor

The performance of the ECG sensor was assessed in two studies, comprising 16 adults (Study 1) and 5 infants (Study 2). For both samples, RSA values derived from LittleBeats™ IBI data showed expected changes as a function of task demands (i.e., baseline versus cognitive challenge for adults; baseline and play episode versus still episode of the Still Face Paradigm for infants), and those changes mirrored the pattern observed for RSA values derived from BIOPAC data. Further, based on prior work suggesting MAPE values under 10% offer an acceptable degree of error for ECG-related measurements [83,84,85], Study 1 results indicate that the LittleBeats™ ECG signal yielded IBI values that showed acceptable agreement with a gold-standard ECG monitor (MAPE ≤ 5.97%). Although these comparisons showed acceptable levels of agreement among the Study 1 adult sample, performance was substantially higher and more consistent among the Study 2 infant sample (MAPE ≤ 1.66%). 

Differences in performance between the adult and infant samples could be due to age, in that adults are more mobile than young infants, and their data may be more susceptible to movement artifacts. We controlled for this possibility, however, by monitoring ECG when both adults and infants were seated. A more likely explanation is that data were collected on different versions of the device firmware, and the presence of missing samples in the ECG data only occurred in Study 1 (Version 1 firmware). Missing samples were corrected via a custom filtering/editing script, although pockets of misalignment of LittleBeats™ and BIOPAC IBI data were more frequent in these data. Such misalignment may result in higher error rates in the IBI data collected in Study 1 compared with Study 2, yet we underscore that both studies showed the expected patterns of RSA change across challenge versus baseline sessions. Taken together, this pattern of results suggests that a modest level of disagreement in the IBI data (likely due to some misalignment) for the adult sample did not impact the measurement of cardiac vagal tone via RSA. Lastly, results from the infant data collected with Version 2 of the firmware (i.e., no missing samples), although not showing an absolute 1:1 agreement with BIOPAC, indicate that the LittleBeats™ platform is a promising sensor for capturing IBI data for infants under 12 months of age. 

### 8.2. IMU Sensor

Turning to the validation of the IMU, although the classification of four activities (i.e., upright, walk, glide, squat) among an adult sample showed a higher performance using accelerometer data from the smartphone, the performance on LittleBeats™ data was also high (e.g., F1-score = 88%; kappa = 0.79), and the discrepancy in F1-score was less than 4 percentage points. The smartphone acceleration values go through additional filtering via the smartphone’s internal software, which likely improves performance. LittleBeats™ does not go through such processing, and thus, performance may increase with the additional postprocessing of LittleBeats™ data and more complex algorithms [58], including various filtering (e.g., Butterworth [102], Savitzky Golay [103]) and smoothing techniques. In summary, Study 3 results indicate that the IMU data of LittleBeats™ are stable and preserve similar information as an off-the-shelf mobile platform, i.e., a Google Pixel 1 smartphone.

### 8.3. Audio Sensor

Lastly, audio data were assessed in two studies: speech emotion recognition (SER) among 8 adults (Study 4) and automatic speech recognition (ASR) among 12 adults (Study 5). Performance on the SER task did not differ significantly between the two devices, suggesting that the LittleBeats™ audio performed as well as the smartphone audio. The signal from LittleBeats™ resulted, however, in slightly less accurate ASR than the smartphone signal (Study 5). This contrast in findings between Studies 4 and 5 may be the result of the types of acoustic features used in these two classifiers. ASR depends on the accurate characterization of individual phonemes and may, therefore, suffer from minimal amounts of missing data (i.e., 2.3% in Study 5) in the LittleBeats™ data. Speech emotion recognition, on the other hand, classifies a vector composed of thousands of partially redundant long-term signal features, each of which characterizes the trajectory or statistics, over time, of one or more low-level signal descriptors. It is likely, therefore, that the redundancy built into the emotion classifier’s feature extraction algorithms permits LittleBeats™ data to achieve accuracy levels equivalent to the smartphone data despite a modest degree of missing samples (i.e., 8.7% in Study 4). Our ultimate aims, speaker diarization and vocalization labels for infants (crying, fussing, babbling) and family members (e.g., infant-directed speech, laughter, singing; see [43,67]), resemble speech emotion recognition more than they resemble ASR. Only ASR requires the correct recognition of phoneme-length segments (10–100 ms) based directly on low-level signal descriptors, whereas SER and our tasks of interest require correct classification of one- or two-second speech segments on the basis of the segment-length trajectories and statistics of low-level descriptors.

It is important to note that overall performance on the ASR task was high despite the difference in performance observed for LittleBeats™ versus smartphone data, whereas performance on the SER task was modest yet similar across LittleBeats™ and smartphone data. Because emotions can be expressed in different ways and with different intensities, we attribute the overall modest performance on SER to the difficulty of the task. Unlike the prior RAVDESS study [93], we did not use professional actors. Thus, we were challenged to obtain a reliable adult emotional speech corpus, although we took this challenge into consideration by conducting a cross-validation check on our corpus and selecting four basic emotions with human interrater agreement to conduct our experiments. For the purpose of this validation study, however, what is most germane is that the SER algorithm performed equally well when using audio from the LittleBeats™ device and the smartphone.

### 8.4. Limitations and Future Directions

The preliminary technical validation studies reported here provide initial evidence that the quality of LittleBeats™ sensor data are largely comparable to data obtained from gold-standard devices. Nonetheless, we note several limitations. First, samples sizes were relatively small, especially for Study 2, in which our validation of the ECG sensor was conducted with five infants. We currently have a larger validation study of the ECG sensor underway among infants between 3 and 10 months of age, in which we aim to further compare the quality of the LittleBeats™ ECG and IBI data against the BIOPC gold-standard equipment in the lab. 

Second, for the IMU and audio data, we recruited adult samples with whom we could implement standardized laboratory tasks and use algorithms that have been previously validated for these data types. Because the features of infants’ vocalizations, postures, and physical movements may be qualitatively different than adults in some ways (e.g., higher pitch range), it is imperative to further validate the LittleBeats™ platform using data collected among infant samples. Yet, because standardized controlled tasks cannot be readily carried out to assess infant vocalizations or movements and because we know of no well-established algorithms to assess infant vocalizations or movements, our best opportunity to validate the LittleBeats™ IMU and audio data among infants is via analytic validation approaches. To this end, we are developing new algorithms, applying them to the LittleBeats™ sensor data, and assessing their performance against ground truth labels provided by trained human annotators. The analytic studies under way will add to our related prior work on the detection and labeling of infant vocalizations and sleep/wake states [43,44,67]. 

Third, the technical validation studies were conducted using brief controlled laboratory tasks. Because we ultimately aim to use LittleBeats™ to assess infant functioning in naturalistic contexts (i.e., home) and across long periods of time (i.e., daylong recordings that last 8+ h), validation efforts must also consider these factors. Indeed, in our ongoing analytic validation of infant data, including ECG data, we include data collected during semi-structured tasks in the lab or remotely with researchers present (parent–infant play session), as well as daylong recordings made in the home without researchers present. With respect to daylong audio recordings in the home, we place high priority on participant privacy and data confidentiality (see [45] for our prior findings related to parents’ perspectives on using LittleBeats™ in the home). Such usability issues go hand in hand with technical and analytic validation efforts, and we continually assess participants’ experiences and concerns as we work to implement best practices that increase usability and data security and minimize participant concerns about data privacy [24].

## 9. Conclusions

Taken together, the results provide confidence in the quality of data obtained from the LittleBeats™ ECG, motion, and audio sensors. Although we have designed the LittleBeats™ platform for use with infants and young children, we focused our technical validation on predominantly adult samples because, by doing so, we are able to validate the device using structured laboratory tasks for which well-established performance measures exist, and for which the performance of this device can be compared to the performance of gold-standard devices used in prior research. This technical validation is an important step in the validation of the platform. A key advantage of LittleBeats™ is the integration of multiple sensors into one platform. We are currently leveraging this multimodal capability, in combination with the further development of postprocessing pipelines, to further increase data quality and, in turn, the performance of algorithms designed to automatically detect infant/child behaviors and physiological states (e.g., sleep/wake detection [44]). 

## Figures and Tables

**Table 1:** Error statistics and Bland–Altman analyses for adult participants’ (Study 1) interbeat intervals (in milliseconds) during the baseline, puzzle, recovery, and matrices tasks.

Session (*n* Observations)	Absolute Mean Error	MAPE (%)	Mean Error (*SD*)	Bland–Altman Analysis	
				Lower LoA	Upper LoA
Baseline (*n* = 3355)	49.6	5.93%	11.1 (77.3)	−162.54	140.33
Tangram puzzle (*n* = 3744)	41.9	5.29%	4.5 (62.8)	−127.59	118.65
Recovery (*n* = 2777)	49.7	5.97%	12.7 (73.1)	−156.02	130.55
Matrices (*n* = 4589)	45.6	5.62%	10.6 (68.3)	−144.51	123.28

Note. Except for MAPE, which is reported as a percentage, all other values are reported in milliseconds. Mean error computed as BIOPAC minus LittleBeats™ IBI. MAPE = mean absolute percent error; LoA = 95% limits of agreement.

**Table 2:** Error statistics and Bland–Altman analyses for infant participants’ (*N* = 5, Study 2) interbeat intervals (in milliseconds) during the baseline and SFP play, still, and reunion episodes.

Session (*n* Observations)	Absolute Mean Error	MAPE (%)	Mean Error (*SD*)	Bland–Altman Analysis	
				Lower LoA	Upper LoA
Baseline (*n* = 907)	5.4	1.17%	1.3 (7.22)	−15.45	12.84
SFP play episode (*n* = 1075)	4.4	0.96%	2.0 (6.58)	−14.87	10.93
SFP still episode (*n* = 936)	6.9	1.66%	1.7 (10.93)	−23.09	19.75
SFP reunion episode (*n* = 1472)	5.6	1.22%	1.7 (9.29)	−19.92	16.49

Note: Except for MAPE, which is reported as a percentage, all other values are reported in milliseconds. Mean error computed as BIOPAC minus LittleBeats™ IBI. MAPE = mean absolute percent error; LoA = 95% limits of agreement.

**Table 3:** Confusion matrices of activity classification (4 classes, 1254 samples) with LittleBeats™ and smartphone IMU data.

	LittleBeats™ Data				Smartphone Data			
Ground Truth Labels	UP	WA	GL	SQ	UP	WA	GL	SQ
Upright (UP)	805 (0.991)	3 (0.004)	1 (0.001)	3 (0.004)	803 (0.989)	0 (0)	7 (0.009)	2 (0.002)
Walk (WA)	45 (0.300)	88 (0.587)	8 (0.053)	9 (0.060)	1 (0.007)	142 (0.947)	3 (0.020)	4 (0.027)
Glide (GL)	2 (0.011)	11 (0.063)	146 (0.830)	17 (0.097)	6 (0.034)	1 (0.006)	150 (0.852)	19 (0.108)
Squat (SQ)	9 (0.078)	13 (0.112)	17 (0.147)	77 (0.664)	0 (0)	8 (0.069)	40 (0.345)	68 (0.586)

Note: Rows represent the ground truth labels (812 for upright, 150 for walk, 176 for glide, 116 for squat), and columns represent the predicted data. The proportions of a given ground truth label that were predicted as upright, walk, glide and squat, respectively, are shown in parentheses.

**Table 4:** A 2 × 2 Chi-square contingency matrix comparing correct and incorrect classification on an activity recognition task using data from LittleBeats™ and a smartphone (Study 3).

		LittleBeats™	
		Correct	Incorrect
Smartphone	Correct	1065	95
	Incorrect	61	33

Note: The number of samples reported in each cell are the summation of ten different sets of test data randomly selected during each fold.

**Table 5:** Confusion matrices of speech emotion recognition (4 classes) with LittleBeats™ and smartphone audio data.

	LittleBeats™ Data				Smartphone Data			
Ground Truth Labels	NEU	HAP	SAD	ANG	NEU	HAP	SAD	ANG
Neutral (NEU)	22 (0.786)	2 (0.071)	2 (0.071)	2 (0.071)	17 (0.607)	1 (0.036)	6 (0.214)	4 (0.143)
Happy (HAP)	0 (0)	24 (0.649)	2 (0.054)	11 (0.297)	1 (0.027)	25 (0.676)	2 (0.054)	9 (0.243)
Sad (SAD)	6 (0.154)	8 (0.205)	25 (0.641)	0 (0)	6 (0.158)	4 (0.105)	27 (0.711)	1 (0.026)
Angry (ANG)	0 (0)	6 (0.158)	4 (0.105)	28 (0.737)	1 (0.026)	12 (0.316)	3 (0.079)	22 (0.579)

Note: Rows represent the ground truth labels, and columns represent the predicted data. The proportions of a given ground truth label that were predicted as neutral, happy, sad, and angry, respectively, are shown in parentheses.

**Table 6:** 2 × 2 Chi-square contingency matrix comparing correct versus incorrect classification on speech emotion recognition using data from LittleBeats™ and smartphone (Study 4).

		LittleBeats™	
		Correct	Incorrect
Smartphone	Correct	75	16
	Incorrect	23	27

**Table 7:** Word error rates (WER) for LittleBeats™ and smartphone audio with and without a language model (Study 5).

Models	LittleBeats™ WER	Smartphone WER
Greedy decoding	5.75%	3.58%
Beam search	5.80%	3.63%
Beam search + language model	4.16%	2.73%

## Data Availability

Data presented in Studies 1, 2, and 3 will be made publicly available. Audio data presented in Studies 4 and 5 will not be made available due to their identifiable nature, but all secondary data files and code will be made publicly available.

## Supplementary Materials

The following supporting information can be downloaded at: https://www.mdpi.com/article/10.3390/s24030901/s1.
