# Supplementary material for: Preliminary Technical Validation of LittleBeats™: A Multimodal Sensing Platform to Capture Cardiac Physiology, Motion, and Vocalizations
Source: Sensors (Basel). 2024 Jan 30;24(3):901. doi: 10.3390/s24030901 (PMC10857055; doi:10.3390/s24030901)

Table S1.

Summary of Software and Algorithms used for the LittleBeats™ Platform and in the Technical Validation Studies

Paper section	Software/open-sourced ML model used	Purpose
Device overview	Code developed in-house in C using Adafruit Libraries	LittleBeats data acquisition
Device overview	Python code developed in-house	To pre-process the data before running the ML/signal processing algorithms
Studies 1 & 2	CardioPeak & Segmenter	To extract IBI (also called R-R peaks) from ECG data for a given session or task
Studies 1 & 2	CardioEdit v1.5	To manually edit IBI data
Studies 1 & 2	CardioBatch Plus	To extract respiratory sinus arrythmia (RSA) values from the IBI data files
Study 3	Sklearn	To implement ML Algorithms
Study 4	Built-in clipfix function in Audacity software	To alleviate clipping in LB audio
Study 4	sklearn	To implement LDA algorithm to perform SER
Study 5	Wav2vec 2.0	To perform ASR on Rainbow Passage
Study 5	KenLM	To implement language modeling used in ASR inference

Figure S1. Plots of aligned interbeat intervals (IBI) from LittleBeats™ (orange line) and BIOPAC (blue line) during (a) baseline, (b) puzzle task, (c) recovery, and (d) matrices task from an adult participant (Study 1). The X and Y axes each represent time in seconds.

(a) Baseline

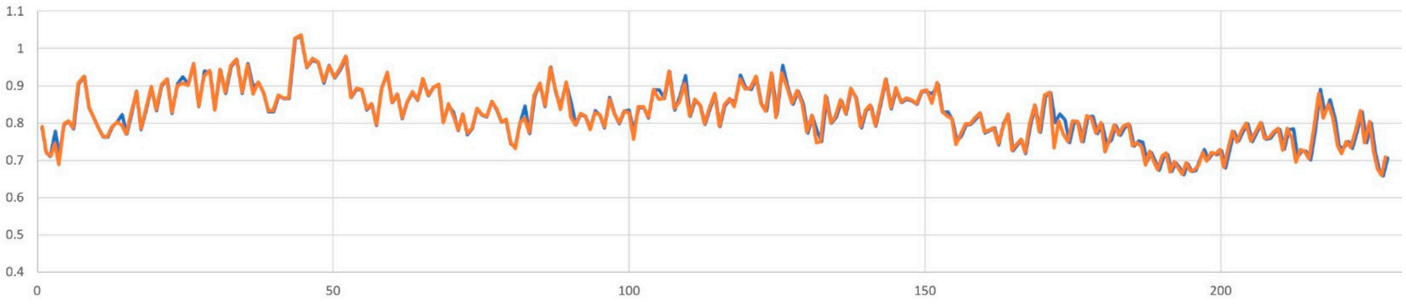

(b) Puzzle task

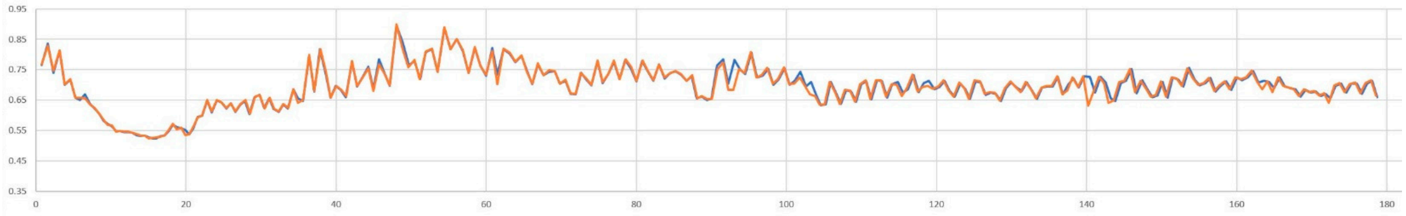

(c) Recovery

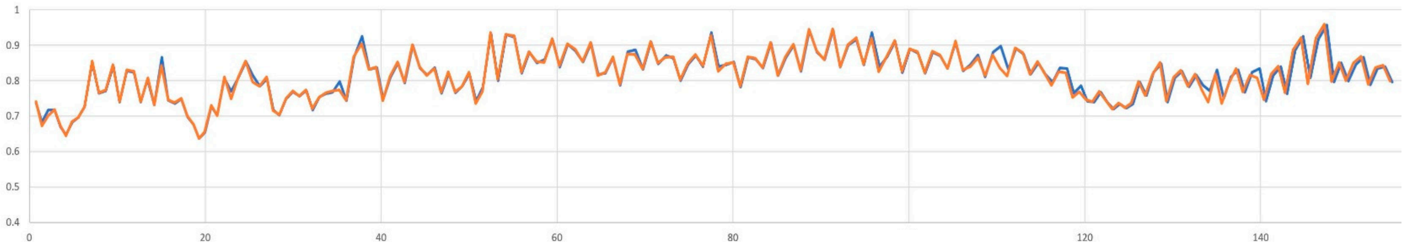

(d) Matrices task

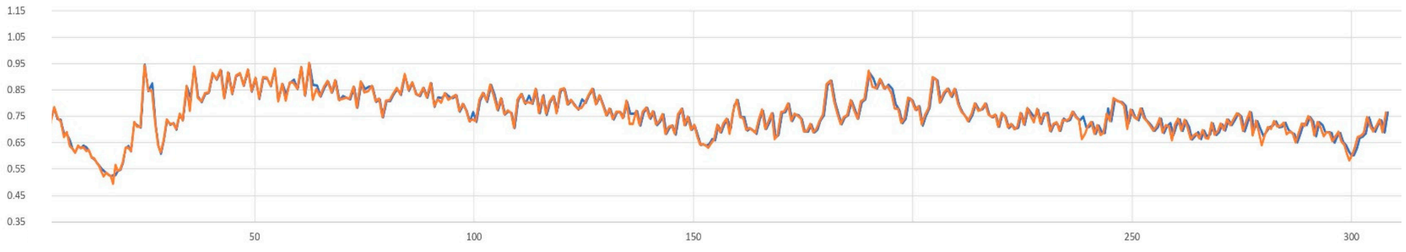

Figure S2. Plots of aligned interbeat intervals (IBI) from LittleBeats™ (orange line) and BIOPAC (blue line) during (a) baseline, (b) SFP play, (c) SFP still, and (d) SFP reunion episodes from an infant participant (Study 2). The X and Y axes each represent time in seconds.

(a) Baseline

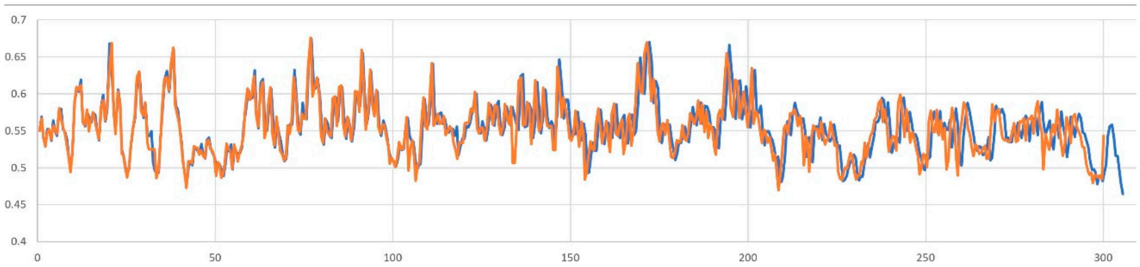

(b) SFP play

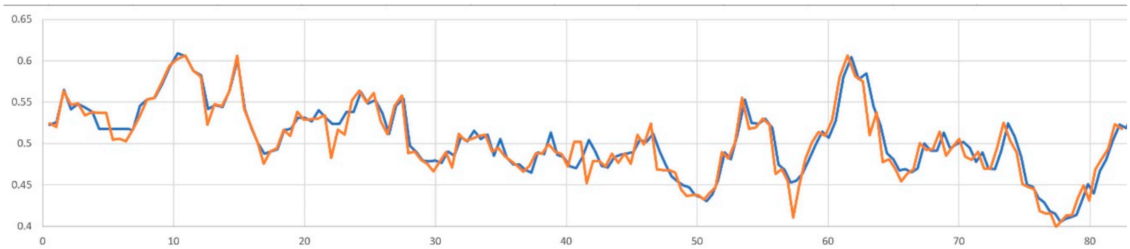

(c) SFP still

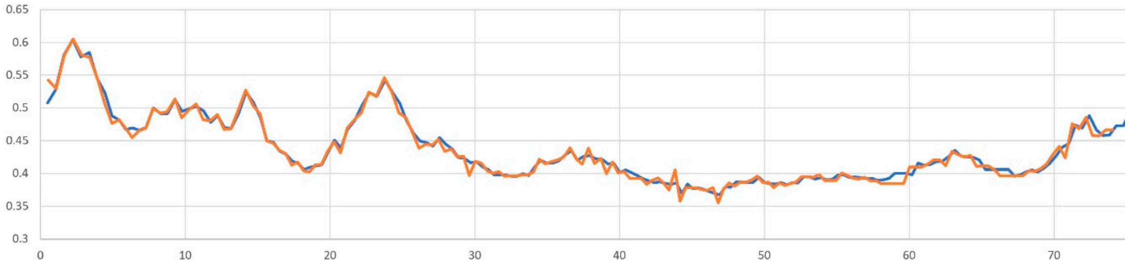

(d) SFP reunion

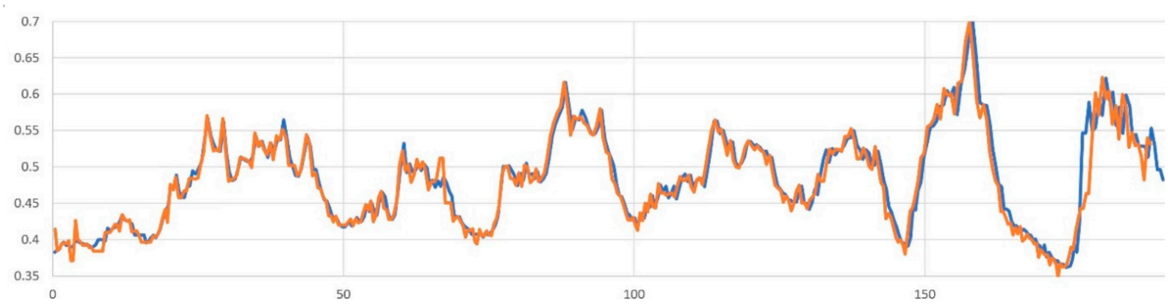

Supplement: Supplementary file 1 [file sensors-24-00901-s001.zip › sensors-2573990-supplementary.pdf]
